# The Relevance of Gender in Tumor-Influencing Epigenetic Traits

**DOI:** 10.3390/epigenomes3010006

**Published:** 2019-01-28

**Authors:** Victoria Sarne, Sandrina Braunmueller, Lisa Rakob, Rita Seeboeck

**Affiliations:** Department Life Sciences, IMC University of Applied Sciences Krems, 3500 Krems an der Donau, Austria

**Keywords:** epigenetics, gender medicine, DNA methylation, tumor marker, sex, carcinogenesis, precision medicine

## Abstract

Tumorigenesis as well as the molecular orchestration of cancer progression are very complex mechanisms that comprise numerous elements of influence and regulation. Today, many of the major concepts are well described and a basic understanding of a tumor’s fine-tuning is given. Throughout the last decade epigenetics has been featured in cancer research and it is now clear that the underlying mechanisms, especially DNA and histone modifications, are important regulators of carcinogenesis and tumor progression. Another key regulator, which is well known but has been neglected in scientific approaches as well as molecular diagnostics and, consequently, treatment conceptualization for a long time, is the subtle influence patient gender has on molecular processes. Naturally, this is greatly based on hormonal differences, but from an epigenetic point of view, the diverse susceptibility to stress and environmental influences is of prime interest. In this review we present the current view on which and how epigenetic modifications, emphasizing DNA methylation, regulate various tumor diseases. It is our aim to elucidate gender and epigenetics and their interconnectedness, which will contribute to understanding of the prospect molecular orchestration of cancer in individual tumors.

## 1. Introduction

Epigenetic traits, like DNA methylation, can strongly influence a tumor’s behavior and vulnerability. So does also patient’s gender. Those two fields of research, epigenetics and gender medicine, had a rather exotic standing in oncological research until their importance was clearly shown in the last couple of years [1,2,3,4]. Within this review, we aim to give an overview about the most influential aspects of gender medicine and epigenetic DNA methylation on carcinogenesis and tumor development, alluding also to diagnostics and therapy. Furthermore, we feature the crossing points of gender research and epigenetics in order to complete the current view of this promising research area.

Gender medicine has roots in the feminist movement, balancing the well-settled male-dominated view in medicine. But gender medicine is not only about establishing equality among female and male patients; it is also about considering the social gender of an individual. Thus, gender medicine is always individualized medicine, respecting the individual patient’s sex, age, ethnicity, education, and social as well as environmental influences. While some implications in oncological processes of, especially biological, gender are well described, many questions still have to be addressed and will find answers considering all aspects of gender. A basic genomic view reveals that while the Y chromosome carries mainly genes associated with sexual function, the X chromosomes harbor genes coding for physiological processes of the heart, brain and immune system. As a result, women, carrying two copies of the X chromosome, although one of them is silenced, have a stronger immune system than men, a lower risk for infectious disease and better antibody production following vaccination [5,6]. On the other hand, women are also more frequently affected by autoimmune disease like multiple sclerosis and rheumatic disease [5,7]. Interestingly, cytochrome p450 isoforms, playing a critical role in drug metabolism and clinical effectiveness, are differentially expressed in female versus male patients [8,9]. Lately, an interconnectedness with epigenetics was reported in two independent in vitro studies [10,11]. Furthermore, elevated CYP1A1 levels are detected in female lung cancer (LC) patients with a smoking history, correlating to a lower DNA repair capacity and increased risk of developing smoking-related p53 mutations [12]. A significantly higher expression of CYP27B1 was found in tumors of the distal colon in male patients compared to female ones. Rectal tumors, however showed a higher expression of CYP27B1 in women only [13].

In oncology, a gender disparity in incidence, invasiveness as well as the associated prognosis has been observed for various cancer entities. The scientific community gains more and more knowledge, emphasizing that patient gender is still underestimated in clinical practice for the treatment of the major types of cancer.

In mammalian genomes, DNA methylation studies have focused on the covalent addition of a methyl residue to the fifth carbon of a cytosine (5meC) nucleotide, which is almost exclusively situated 5’ to a guanine nucleotide [14]. Besides, methylation of 5meCpA dinucleotide can also occur in the mammalian genome but is usually restricted to pluripotent cells in embryonic development [15]. DNA methylation triggers chromatin condensation in the dynamic conformation of nucleosomes, which frequently associates with gene silencing [16].

DNA methylation is catalyzed by DNA methyltransferase (DNMT) enzymes which can either maintain methylation or create de novo methylation. In the latter, cytosine-phosphate-guanine (CpG) loci are methylated without the presence of a template strand containing 5meCpG. This process is catalyzed by the methyltransferases DNMT3a and DNMT3b. These enzymes are primarily active during early embryonic development but were also observed in adult mice brain cells to be involved in learning and memory [17,18]. In contrast to that, DNMT1 is responsible for maintenance methylation, which takes place on daughter strands after DNA replication and requires the presence of the methylated template to pass the methylation pattern to the next cell generation [19,20].

The presence of physiological differences between males and females is strikingly obvious. Despite that, differences in the epigenome might not be as clear. Even though some studies have reported no difference in the autosomal DNA methylation between genders, a lot of other have found differentially methylated genes (DMGs) as well as differentially methylated CpG sites [21,22,23,24,25].

Generally, a trend towards higher overall methylation in males was found and blood global long interspersed nuclear elements-1 (LINE-1) methylation is also significantly higher in males than in females [2,5,7,8,9,26,27]. If the same is true in disease, whether this has an influence on the response to treatment and disease outcome needs to be further evaluated. Two studies recently investigated differentially methylated CpG sites in the umbilical cord blood of boys and girls. Overall, most differentially methylated CpG sites were more methylated in girls than in boys and when looking at the associated genes, they play a role in nervous system development, brain and heart tissue, as well as behavior [28,29]. Interestingly, not only the differentially methylated regions (DMRs) located on the X chromosome are methylated to a greater extent, as would be expected due to X chromosome inactivation, but also autosomal DMRs followed this trend [28]. In adolescents the global methylation level of leukocyte DNA has been found to be significantly higher in females than males [30]. In a reduced representation bisulfite sequencing (RRBS) analysis of human adult liver samples, 460 tiles were differentially methylated and overall, mean methylation was again significantly higher in females compared to males [31]. The analysis of human fetal liver samples, where the fetuses have not been exposed to cigarettes, revealed that DNMT1 expression is higher in males than females, while the opposite is true for the expression of DNMT3B [32]. In concordance with this finding, the expression of DNMT3B was significantly higher in adult liver samples of females than males [33]. Blood DNA of males had lower Alu methylation but higher LINE-1 methylation compared to females [34]. Global methylation was significantly higher in white blood cell DNA of females compared to males. Smoking reduced the global methylation in both sexes, even though the effect was stronger in women [35]. The analysis of peripheral blood cells revealed that the promoter region IV of the *BDNF* gene is significantly more methylated in females than in males [36]. Although several candidate genes have been proposed, a meta-analysis comprising 81 studies found only the gene *MGMT* to possess significant sex-specific DNA methylation, with all *MGMT* probes being more methylated in females [25]. Despite that, other studies have reported the opposing trend, *MGMT* being more methylated in males [22,37]. From the present data it can be concluded that there are, indeed, physiological differences in the epigenome of males and females. Nonetheless, more research is this area is required.

Genomic imprinting is a phenomenon in which epigenetic mechanisms lead to parental-specific gene expression in a diploid cell. This process affects both male and female offspring and is, therefore, considered to be a consequence of inheritance, not sex [38]. Nevertheless, differences between sexes in the DNA methylation of imprinted genes have been found. In a study with a large European cohort, autosomal DNA methylation levels between men and women were compared. The study revealed 1184 CpGs which showed stable DNA methylation differences between sexes. These sites were found to be enriched at imprinted genes [39]. Sex-specific changes in DNA methylation were also found in offspring after suffering nutritional insult during pregnancy [40].

Another important epigenetic factor to consider are histone modifications, which will not be covered in this review. Recent literature includes [41,42,43,44,45].

## 2. Gender, DNA Methylation and Cancer

Worldwide, ranked by incidence as well as mortality, LC is the most common cancer [46,47]. In terms of incidence, LC is followed by breast cancer (BC), colorectal cancer (CRC) and cancers of prostate, stomach and liver. These six cancer entities together represent more than half of the global incidences in 2018. As for mortality, LC alone is responsible for 18.4% of 2018th tumor deaths, followed by CRC, stomach, liver and BC [46,47]. Gender-specific differences in incidence, progression, treatment and survival have been reported for a variety of cancer types, hence the most common types of cancer differ between sexes. In men, the five most common cancers are: lung, prostate, colorectal, stomach, and liver. In women the most common cancer is breast, followed by colorectal, lung, cervix uteri and stomach. In cancers affecting both genders, a general higher risk as well as absolute number of incidences and deaths accounts for the male population, although there is great variance among specific entities and geographical regions [46,47].

In this review we focus on solid tumors. As for hematological malignancies we recommend the review by Ben-Batalla et al. published in late 2018, which discusses the sexual dimorphism in non-solid cancer in more depth than the scope of this review allows [48].

Aberrant epigenetic regulation is known to directly contribute to disease specific phenotypes. In this fashion, epigenetic changes are involved in carcinogenesis and the development of tumor disease [49]. Among other epigenetic mechanisms, DNA methylation seems to play a particularly important role in the initiation and progression of cancer. Cancer cells often display methylation patterns that differ from their non-cancerous counterparts [1]. Both hyper- and hypo-methylation have been proposed to be important events in carcinogenesis and immune signaling in tumor tissue [50]. As described above, hypermethylation of cytosine in CpG islands (CGIs) by DNMTs of promoter regions has been shown to enable gene repression. Therefore, hypermethylation in tumor suppressor genes (TSGs) potentiates tumorigenic activity due to the disruption of critical cellular processes like cell cycle control, DNA repair and apoptosis [51,52,53].

Even though it is well established that epigenetic modifications play a role in cancer progression in many different types of cancers and are even thought to drive metastasis, there is only little information available about gender-associated differences in epigenetic patterns within the different cancer types [54,55,56,57,58,59,60]. Epigenetic inactivation can be viewed as an alternative to genetic mutations in cancer [61]. The change in phenotype subsequently facilitates the adaption of cancer cells to their specific environments [61]. In fact, it has been suggested that heritable losses of gene function may be mediated as often by epigenetic as by genetic abnormalities [62,63]. Looking at the vast amount of data available, it is becoming increasingly apparent that instead of cancer being either a genetic or epigenetic disease, the synergy between these two processes drives tumor progression from the earliest to latest stages of disease [64]. A prominent example is KRAS mutation upon epigenetic stimulation triggered by long-term exposure of cells to cigarette smoke [65]. These changes include initial repressive polycomb marking of genes, followed by the later manifestation of aberrant DNA methylation. Epigenetic changes may, therefore, potentially prime cells for oncogene addiction [66].

Several studies have identified a variety of DMRs in different cancers. Specific DMRs have been proposed to not only be tumor specific, but also subtype specific. Subsequently, DNA methylation profiling has confirmed the existence of epigenetic subtypes in cancers. In this fashion, specific methylation profiles of LC samples identified subtypes of tumors with distinct prognoses [50]. Similarly, a differentiation between tumor and control lung tissue, as well as identification of novel DMRs for the two most common non-small cell LC (NSCLC) subtypes, adenocarcinomas and squamous cell carcinoma is reported based on methylation patterns [67]. DNA methylation subgroups, referred to as epitypes, may discriminate between tumor subtypes like neuroendocrine tumors (SCLC and LCNEC) and adenocarcinoma [50,67,68,69].

Lung cancer is of prime interest for gender research, as it is strongly influenced in pathogenesis as well as progression by hormones and the mutational status of their receptors and possesses several further gender-dependent characteristics. Global as well as national reports show a dramatic, gender specific change in LC incidences and death rates [70,71,72,73]: while historically men were more frequently affected by LC, the number of women suffering from LC is increasing quickly, i.e., by 30% in the last 10 years in the exemplary Austrian population [72]. At the same time, mortality rates are increasing for women only, which indicates dramatically that LC treatment is obviously a gender topic [72]. Also, in tumor subtypes gender-differences are known, NSCLC is more common in men, however there was an increase in the proportion of women <55 years that has manifested throughout the last decade. Adenocarcinoma is rather predominant in women, while squamous cell carcinoma (SCC) is more likely to develop in men >72 years [74]. Smoking is the major risk factor for developing LC, therefore the male to female ratio of relative risk (RRR) was found to be 1.61 compared to non-smokers in a meta-analysis of 47 publications. Currently smoking men have a higher risk of developing LC than women, regardless of smoking quantity and duration or years since quitting [75]. Interestingly, the absolute risk of LC in never smokers was higher in women than in men, while smoking men have a higher risk of developing LC compared to smoking women [76].

### 2.1. Hormonal Influences, Microsatellite Instability (MSI) and Chromosomal Instability

Reasons for carcinogenic differences between female and male LC patients are not clear. In LC it was found that progesterone receptor (PR) expression in tumor stromal cells correlated with improved disease-specific survival (DSS) for both genders, while PR expression in tumor epithelial cells was associated with poor prognosis in females only and could therefore be used as prognostic biomarker [77]. An important role in the pathogenesis of NSCLC is taken over by estrogens; its receptor, estrogen receptor (ER) β is predominantly found in ovary and lung tissue and ERα is rather found in breast, ovary and endometrial tissues [78]. ERβ is expressed in NSCLC tumors of both sexes, whereas a higher level was found in male individuals. More precisely, women were 46% less likely to have ERβ-positive tumors than men [79]. Estrogen signaling causes proliferation in NSCLC cell lines, and recently progesterone, was found to be expressed in correlation with cell proliferation. In combination, these two stimulate vascular endothelial growth factor secretion in vitro, and thereby promote cancer-associated angiogenesis and tumor progression [80]. *ERα* is significantly more methylated in males than in females suffering from LC [81]. Considering chromosomal instability, polymorphisms of *GSTT1* and *GSTM1*, two Glutathione S transferases important for detoxification, were found to result in an increased LC risk. Specifically, the *GSTT1* null genotype was associated with an increased risk in men only, while *GSTM1* null genotype lead to an increased risk in female subjects [82]. Another marker for NSCLC is *EML-ALK4*. This fusion oncogene was found to be associated preferentially with male gender, never/light smoking behavior and younger age [83]. The clinically most important marker to date is the mutational status of the epidermal growth factor receptor (EGFR), which is a glycoprotein found in high density on the NSCLC cell surface, genetic mutations of which result in uncontrolled stimulation of cell proliferation. Those mutations are found at higher frequency in women [84]. This fact was also confirmed in the Iressa Pan Asia Study (I-PASS), where EGFR tyrosine kinase inhibitor (TKI) therapy was confirmed [85].

Dependencies on estrogen and androgen are major gender-associated factors, influencing essentially all tumor entities. In CRC AR, ERα and ERβ, together with the membrane receptors of growth-inhibiting melatonin MT1 and MT2, were observed to be downregulated in the early stage and advanced tumors in male patients only [86]. The overall expression of MT1 and MT2 correlated positively with AR, ERα and ERβ in men and with ERα and ERβ in women [86]. Aggressive, right-sided colon cancer is frequently associated with microsatellite instability (MSI), which might develop at an increased risk in older women due to a lack of estrogen hormone and can be counteracted by hormone replacement therapy [87]. *hMLH1*, which is analyzed for MSI is, together with *p14^ARF^*, significantly more methylated in female than in male CRC patients [88,89]. Genetic differences were found in CRC patient samples analyzing chromosomal copy number aberrations, were female gender associated with significantly higher numbers of gains in chromosome arms 1p21.2-q21.3, 4q13.2, 6q11.2 and decreased copy numbers of 11q25. Almost half of the male samples displayed a “feminization” phenomenon, as they gained a whole or an arm of an X chromosome and/or lost the Y chromosome. This phenomenon was associated with microsatellite-stable CRCs and wild-type *BRAF* cancers [90]. A link between androgen receptor (AR) status, poor survival and the expression of TUBB3/TUBB6 was identified in female CRC samples only. In males, however, there was no relationship between TUBB3/TUBB6 expression and the outcome and aggressiveness of cancer [91].

In gastric cancer (GC), MSI is also more common in females and mutated samples [92]. Generally, it is suggested that the presence of estrogens protects females from GC, and hence there is an increase in incidence after the menopausal age [93]. The protective effects of estrogen were also suggested for *H. pylori*-associated GC [94].

In hepatocellular carcinoma (HCC), the incidence rate of male:female patients is approximately 3:1 and was traditionally explained by a gender-dependent differences in exposure to risk factors, such as higher HBV and HCV infection rates amongst males, as well as higher alcohol consumption, smoking behavior and increased iron stores [95]. However, more recent studies emphasize that androgens influence HCC progression and, therefore, might be responsible for the dimorphism, rather than sex-specific risk factor exposure. In this manner, non-environmental endogenous factors might play a role in male susceptibility to HCC, such as higher body mass index (BMI) and higher levels of androgenic hormones [95]. This scheme is further supported by the observation that chronic liver disease progresses into cirrhosis more rapidly in males and therefore leads to HCC development faster [96]. Sex hormones and their receptors were found to greatly contribute to the gender disparity in inflammation-driven HCC. Generally, androgens were found to exert tumor-promoting functions, whereas estrogens were found to possess tumor-inhibiting potential [97].

BC is a clear gender-dependent tumor entity with only 1% of all BC cases in the US being male BC (MBC) [98]. Nevertheless, sex specific differences in clearance, general incidence rates as well as pathology have been reported [99]. Looking at pharmacokinetics, it was found that the clearance of the chemostatic drug Doxorubicin was significantly higher in male patients, compared to females, which could be due to epigenetic regulation of cytochrome family members [100].

BC with *BRCA1* or, more commonly, *BRCA2* mutations differs in its pathological characteristics between MBC and female breast cancer (FBC). *BRCA2* mutated MBC was suggested to possess generally greater biological aggressiveness [101]. The biology of MBC is thought to resemble late-onset FBC. However, MBC was also found to occur later in life and with a higher proportion of *ER* positive and *PR* positive tumors [102].

In familial BC, similarly to LC, males shower higher levels of global hypermethylation than the female cohort [103]. In a recent in-vitro study female hormone-free, ER-/PR- positive BC cells were assessed and revealed a dependency of ERα expression on PR expression. PR was shown to directly bind ESR1 locus preserving a low DNA methylation and expression of Eα [104]. This mechanism emphasizes the strong impact of ERα promoter methylation status on endocrine therapy and identifies it as a valuable predictive biomarker.

Furthermore, there was also a correlation between *ER* status and *RASSF1A* methylation. Males showed higher methylation of *RASSF1A* when the *ER* was not mutated, while females showed higher promoter hypermethylation levels concurrent with *ER* mutation [103].

### 2.2. RASSF1 and MGMT and Further Differentially Methylated Genes (DMGs)

The aforementioned *RASSF1* is a putative tumor suppressor and a major target of tumor-associated epigenetic dysregulation. It mediates death receptor-dependent apoptosis and contributes to the efficient activation of TP53 by disrupting MDM2 interactions and promoting MDM2 self-ubiquitination in cell-cycle checkpoint control, triggered by DNA damage [105]. Generally, mRNA expression of *RASSF1* transcripts is often lost in LC, where methylation was identified as the major mechanism silencing this gene, while mutations are rare [106]. The promoter region of *RASSF1A* is frequently hypermethylated in many types of cancers [107,108,109,110]. However, only in recent years it was investigated whether gender played a role in methylation frequency of *RASSF1A*. It was found that promoter hypermethylation of *RASSF1A* was higher in male LC patients than in female patients [111]. By contrast, female CRC patients showed significantly higher promoter hypermethylation of *RASSF1A*, than males [112].

*O*^6^-methylguanine-DNA methyltransferase (MGMT) repairs DNA lesions caused by alkylating agents and prevents cell death. As a result, the methylation and consequent loss of *MGMT* expression leads to less repair and increased sensitivity of tumor cells towards alkylating drugs. Methylated promoter methylation indicates a significant survival benefit of patients treated with radiotherapy and temozolomide [113]. Determination of *MGMT* promoter methylation is widely used in clinical routines as a predictive biomarker to assess treatment prospects of glioblastoma with the alkylating agent temozolomide [113]. In LC male non-smokers show a higher frequency of *MGMT* promoter hypermethylation than female nonsmokers. Furthermore, *p53* mutated tumors showed higher levels of *MGMT* methylation in males than *p53* wild-type tumors [37].

Again, contrasting *MGMT* promoter hypermethylation was found to be significantly higher in females than males in a Taiwanese CRC patient cohort [114]. In gastric cancer, also females also showed a higher rate of promoter methylation of *MGMT* together with *hMLH1* and *GSTP1* than males [115].

Apart from the gender differences basted on hormonal signaling and receptor expression, we showed that chromosomal instability, as well as the differential methylation of *RASSF1* and *MGMT* are epigenetic tumormarkers, affecting a broad range of entities. In the following we summarize epigenetic marker genes that are under research or approaching clinical implications for selected or general application in various solid tumors.

In LC, DNA methylation patterns of a number of additional genes have been found to have been altered in cancer tissue [65,116,117,118,119,120,121,122,123]. TSGs such *as RASSF1*, *CDKN2A*, *DAPK*, *APC* and *p14^ARF^* are aberrantly methylated in NSCLC as well as other cancers, namely head and neck cancer (HNC), prostate cancer and cervical cancer [124,125,126,127,128,129,130,131,132].

Genes that are tumor specifically methylated in LC, specifically NSCLC, further include *SPAG6* and *L1TD1*. The methylation was also shown to be involved in the transcriptional regulation in these genes. *L1TD1* additionally has been shown to have tumor growth-suppressing properties and seems to be universally methylated and thereby downregulated in NSCLC [133].

Furthermore, several other known and putative TSGs have been identified that are involved in the pathogenesis of LC and are frequently inactivated by methylation [120,122,134,135]. Namely, methylation was identified as underlying mechanism for the reported frequent RARβ expression in NSCLC [136,137,138].

A candidate TSG, *FHIT* was found to be frequently abnormal in LC, and recently reported to also be frequently methylated in primary NSCLC [139,140,141]. *p16^INK4a^* also has been reported to frequently be inactivated by methylation in NSCLC, and even linked to an early stage in the pathogenesis of LC [120,124].

Aberrant *ANK1B* methylation is highly prevalent in LC and allows the discrimination of tumors by histology and patients’ smoking history: Aberrant *ANK1B* promoter methylation was significantly more prevalent in current and former smokers combined than in never smokers [142].

*ZNF677* was found to be tumor-specifically downregulated by methylation and suggested to have cell growth-suppressing properties in NSCLC [134]. Besides, it was reported that methylation is the major mechanism for inactivating *CDH13* [143]. *ZAR1* has also been shown to be inactivated by DNA methylation specifically in lung tumors [144]. A study comparing neuroendocrine tumors with NSCLC found, that *p16^INK4a^*, *APC* and *CDH13* methylation was higher in NSCLC [65]. Additionally, it was found that *p16^INK4a^* was generally more frequently methylated in NSCLC compared to SCLC [65]. Furthermore, apart from the possible discrimination between tumor types, genes that are differentially methylated between tumors of smokers and never-smokers have been identified [117,145]. Genes, which were found to be differentially methylated, are *LGALS4*, *CXorf38*, *MTHFD2*, *TLL2*, *ALPPL2*, *GFI1*, *MYO1G*, *AHRR*, *ZNF385D*, *IER3* and *F2RL3* [117,145,146]. Differential DNA methylation has also been identified as a marker for prenatal smoke exposure in adults [147]. 

In a CRC, the TSG *p16^INK4a^* was found to be differentially methylated in males and females [148]. Here, females show significantly higher methylation, as the methylation of the CGI 5’ of the *p16^INK4a^* tumor suppressor was found to be 8.8-fold more likely hypermethylated in women than in male subjects. Generally, CGI methylation extent was shown to increase from the rectum to the cecum, where women had a higher percentage of developing tumors in the cecum [148,149,150]. In addition, the female gender is associated with CGI methylator phenotype (CIMP) high status in CRC according to two studies [151,152]. Different cancers show different TSG-inactivating DNA methylation profiles and frequencies [153,154]. The concept of CIMP was originally introduced by Toyota et al., in 1999 and describes the synchronous hypermethylation of multiple gene promoter regions [155]. Since then, CIMP has been reported in various types of cancers, including NSCLC and CRC [155,156].

In NSCLC specifically, chromosome 3p-specific CIMP is a frequent epigenetic event [157]. CIMP status and survival prognosis of NSCLC have also been linked [157]. In this manner, adenocarcinoma cases with CIMP have a poorer prognosis than adenocarcinoma cases without CIMP [156]. CIMP status and prognosis have also been linked in CRC [155].

In GC, the mRNA expression and protein expression of DNMT1 is significantly higher in males than in females, which suggests the presence of epigenetic differences between the sexes [158,159]. Accordingly, the promoter hypermethylation of *HACE 1* and *HOXA11* was significantly higher in males than in females suffering from GC [160,161]. Furthermore, it was found that in chronic gastritis patients’ methylation of *DAPK*, *CDH1*, *THBS1*, and *TIMP-3* is higher in males than females [162]. On the other hand, LINE-1 methylation was higher in female GC patients compared to males [163].

HCC also showed different methylation patterns between the two sexes. The CIMP high type was significantly more frequent in male patients compared to female patients. However, when looking at the gender specific methylation of *p16^INK4A^* contradictory conclusions have been drawn. In one study, *p16^INK4A^* promoter methylation was significantly higher in males than females with HCC, while in another study *p16^INK4A^* promoter methylation was significantly higher in females than males. [164,165].

Table 1 gives an overview of genes that are reportedly differentially methylated in tumors. We verified the impact by comparison with data in the TCGA genomic data commons data portal (https://portal.gdc.cancer.gov) to verify mutational status and especially gender differences, and with methylation data of MethHC (https://methhc.mbc.nctu.edu.tw), where differential methylation across tumor entities is deposited. Consequently, the given references are only representative, evidence-based documents of the genes’ epigenetic and gender impact.

## 3. DNA Methylation as Prognostic Marker

Apart from the fact that tumor specific methylation profiles could aid the early detection of NSCLC and other cancers like prostate and cervical cancer in the future, DNA methylation has additionally been linked with prognosis in NSCLC [126,135,186,187]. Consequently, several studies have suggested that the presence of DNA hyper-methylation in NSCLC might be associated with progression, recurrence and long-term-survival [53,157].

A study comparing low- and high-metastatic NSCLC cells, high metastatic cells showed lower methylated *RNF* promoters and, accordingly, lower RNF111 transcriptional expression levels. *RNF* affects TGF-/Smad signaling and is associated with invasion in NSCLC [183].

Furthermore, *p16^INK4a^* methylation was associated with significantly poorer survival, whereas *CDH1* methylation was associated with significantly better survival in a previous study. The same study also showed that the hypermethylation of multiple genes exhibited a significant differential effect on NSCLC patient survival [123].

The presence of methylation on the promoter region of four genes in particular (*p16^INK4a^*, *CDH13*, *RASSF1* and *APC*) in patients with early stage NSCLC that was treated by means of surgery has been associated with early recurrence [187]. Patients with methylated *ZNF677* was associated with shorter overall survival compared to patients with unmethylated *ZNF677* [134].

*hMLH1* methylation was identified as a common event in NSCLC and may aid in the prediction of recurrence and metastasis of NSCLC patients who accepted post-operative adjuvant cisplatin-based chemotherapy. Therefore, *hMLH1* methylation is considered a biomarker of individualized therapy for NSCLC [179,188].

*EGFR* gene methylation was found to not be influenced by age, gender or smoking status of the patient, but rather found to increase with later stages. *EGFR* methylation may, therefore, be used as an indicator for the stage of cancer tissue malignancy [176].

Patients with early stage NSCLC are still at considerable risk of recurrence and death, even after complete surgical resection. Candidate DNA methylation biomarkers were identified allowing patients at low risk of relapse and those at high risk to be distinguished, which could aid in the decision-making process of further treatment [135].

Among the already identified and routinely implemented epigenetic marker genes is the programmatic promoter DNA methylation of the DNA repair gene *MGMT* in the treatment of glioblastoma by temozolomide (an alkylating agent) [181,189]. *MGMT* repairs the lesion caused by temozolomide and, thereby, prevents the induction of cell death. Methylation and loss of expression of *MGMT* leads to less repair coupled to increased sensitivity of cells to the alkylating agent. Clinical studies showed that glioma patients treated with temozolomide and radiotherapy who have a methylated *MGMT* promoter have a significant survival benefit compared to radiotherapy only. At the same time, lack of *MGMT* promoter methylation reset the prognostic advantage to an absence of significant differences between treatment groups [189].

## 4. Epigenetic Mechanisms as Drug Targets

Targeting epigenetic mechanisms in the treatment of NSCLC and other cancers has been proposed. The metastatic capability of NSCLC is closely associated with DNA methylome alterations [190]. The metastasis-prone phenotype could be reversed in vitro by inhibiting DNMT. Due to this fact, epigenetic modulation seems to be a potential therapeutic approach to prevent metastasis formation [190].

Further information about epigenetic therapies in LC can be found in the recent reviews of Schiffman or Carter [51,191]. Among the currently five different epigenetic agents approved by the United States Food and Drug Administration (FDA), two are DNMT inhibitors and three HDAC inhibitors [192]. The DNMT inhibitors have been shown to possess clinical utility on the treatment of myelodysplastic syndrome and leukemia, whereas the HDAC inhibitors are used in the treatment of rare cutaneous T-cell lymphoma [193]. However, this first generation of epigenetic inhibitors has only shown limited utility due to toxicity and off-target effects [192,194]. In solid tumors, epidrugs have shown very modest anti-tumor efficacy in monotherapy as well was in combination with other therapies. Nevertheless, further generations of epidrugs still are considered promising in advancing cancer treatment.

Only recently, clustered regularly interspaced short palindromic repeat (CRISPR) technology became applicable for the development and in-vitro testing of epidrugs. When introducing epigenetic marks, i.e., DNA methylation patterns as prognostic or predictive tumormarkers, a solid understanding of the molecular background is needed. In 2016 for the first time the CRISPR- associated protein 9 (Cas9) based DNA methylation editing tools were published and kicked off a new era of epigenetic research [195,196]. The CRISPR/Cas9 system was originally identified as a natural immune defense system in bacteria and is now famously implemented in molecular biology translational research as a site-specific genome editing tool. In the case of epigenetic editing, the aim is selectively to modify epigenetic marks, e.g., CpG methylation at a targeted locus. To achieve CRISPR/Cas9-mediated epigenome editing, the main strategy is fusing an inactivated Cas9 protein with an epigenetic effector (epieffector) domain [197]. Inactivation of Cas9 → dCas9 leads to a protein that no longer has nuclease activity but a stable DNA binding domain, that can be targeted using sgRNAs. There are various options for epieffector domains, including VPR/VP64 transactivation domains, the DNA demethylating TET domain or DNMT3A domain [195,196,198,199].

## 5. Conclusions

DNA methylation and patient gender are two general parameters of carcinogenesis and tumor progression. Although there is no doubt about their impact, the underlying availability of solid data is limited. We have put together the currently available literature on this cross-sectional research field, showing a strong implication of patient gender and growing evidence of epigenetic tumormarkers for disease prediction as well as prognosis. It has also become clear that gender-dependent differences in carcinogenesis may be interlinked to epigenetic mechanisms that are themselves dependent on the underlying patient’s biological sex and environmental influences. Today, only *MGMT* promoter methylation is comprehensively implemented in molecular diagnostics, but there are numerous biomarkers in the pipelines to clinical implications. We hope that we can raise awareness for the strong influence of patient gender to be considered at any stage from tumor diagnostics over monitoring therapy design to make epigenetic medicine a truly personalized medicine from the start.

## Figures and Tables

**Table 1:** Aberrantly methylated genes of various tumor types and reported gender difference.

Gene	Cancers	Reported Gender Difference
*ANK1*	Pancreatic cancer [166], lung cancer (LC) [142]	
*APC*	Melanoma [167], Nasopharyngeal carcinoma [168], LC [65]	
*CDH1*	Breast cancer (BC) [169], Cervical cancer [127], Head and neck cancer (HNC) [125], LC [65], Oral cancer [170], GC [162]	GC [162]
*CDH11*	Melanoma [171]	
*CDH13*	BC [143], CRC [172], LC [65], Melanoma [167], Prostate Cancer [173]	LC [174]
*CLDN11*	Melanoma [171]	
*COL1A2*	Melanoma [171]	
*DAPK*	Cervical cancer [127], HNC [125], LC [130], Nasopharyngeal carcinoma [168], Prostate Cancer [173], Gastric cancer (GC) [162]	GC [162]
*EGFR*	BC [175], LC [176]	
*ERα*	LC [81]	LC [81]
*ESR1*	LC [174]	LC [174]
*FHIT*	BC [141], Cervical cancer [177], Liver cancer [178], LC [141]	
*GATA5*	LC [174]	LC [174]
*GSTP1*	GC [115]	GC [115]
*HACE1*	GC [160]	GC [160]
*hMLH1*	BC [169], LC [179], Colorectal cancer (CRC) [88] GC [115]	CRC [88], GC [115]
*HOXA11*	GC [161]	GC [161]
*HOXA9*	Melanoma [171]	
*KCNH8*	LC [180]	LC [180]
*L1TD1*	LC [133]	
*LOX*	Melanoma [171]	
*MAPK13*	Melanoma [171]	
*MGMT*	CRC [112], LC [37], GC [115]	LC [37], GC [115]
*MGMT*	LC [37], Glioblastoma [181]	LC [37], CRC [114]
*p14(ARF)*	CRC [89]	CRC [88]
*p16INK4a*	BC [169], Cervical cancer [127], HNC [125], LC [65], Melanoma [167], Prostate Cancer [173]	CRC [148], HCC [164,165]
*PAX6*	LC [174]	LC [174]
*PTEN*	Melanoma [171]	
*RARß*	LC [65], Melanoma [167], Oral cancer [170], Prostate cancer [126]	LC [180]
*RASSF1*	BC [169], Endometrial cancer [182], LC [65], Melanoma [167], Nasopharyngeal carcinoma [168], Oral cancer [170], Prostate Cancer [173], CRC [112]	CRC [112], LC [111], BC [103]
*RNF*	LC [183]	
*SPAG6*	LC [133]	
*SYK*	Melanoma [171]	
*THBS1*	GC [162]	GC [162]
*TIMP3*	GC [162]	GC [162]
*TNFSF10D*	Melanoma [171]	
*ZAR1*	Bladder cancer [184], Cervical cancer [185], LC [144]	
*ZNF677*	LC [134]	

## List of Abbreviations

AR	Androgen receptor
BC	Breast cancer
CGI	CpG island
CIMP	CpG island methylator phenotype
CpG	Cytosine-phosphate-guanine
CRC	Colorectal cancer
DMR	Differentially methylated regions
DNMT	DNA methyltransferase
DSS	Disease specific survival
EGFR	Epidermal growth factor receptor
ER	Estrogen Receptor
FBC	Female breast cancer
GC	Gastric cancer
HCC	Hepatocellular carcinoma
HNC	Head and neck cancer
I-PASS	Iressa Ran Asia Study
LC	Lung cancer
LINE-1	Ling interspersed nuclear elements-1
MBC	Male breast cancer
NSCLC	Non-small cell lung cancer
PR	Progesterone receptor
RRBS	Reduced representation bisulfite sequencing
RRR	Ratio of relative risk
SCC	Squamous cell carcinoma
TKI	Tyrosine kinase inhibitor
TSG	Tumor suppressor gene
AHRR	aryl-hydrocarbon receptor repressor
ALPPL2	alkaline phosphatase
ANK1	ankyrin 1
ANK1B	ankyrin 1, erythrocytic b
APC	APC, WNT signaling pathway regulator
AR	Androgen receptor
BDNF	brain derived neurotrophic factor
BRAF	B-Raf proto-oncogene
BRCA1	breast cancer 1
BRCA2	breast cancer 2
CDH1	cadherin 1
CDH11	cadherin 11
CDH13	cadherin 13
CDKN2A	cyclin dependent kinase inhibitor 2A
CLDN11	claudin 11
COL1A2	collagen type I alpha 2 chain
CXorf38	chromosome X open reading frame 38
CYP1A1	cytochrome P450 family 1 subfamily A member 1
CYP27B1	cytochrome P450 family 27 subfamily B member 1
DAPK	death associated protein kinase
DNMT	DNA methyltransferase
EGFR	Epidermal growth factor receptor
EML-ALK4	echinoderm microtubule-associated protein-like-anaplastic lymphoma kinase
ER	Estrogen Receptor
ESR1	estrogen receptor 1
F2RL3	F2R like thrombin or trypsin receptor 3
FHIT	fragile histidine triad
GATA5	GATA binding protein 5
GFI1	growth factor independent 1 transcriptional represso
GSTM1	glutathione S-transferase mu 1
GSTP1	glutathione S-transferase pi 1
GSTT1	glutathione S-transferase theta 1
HACE 1	HECT domain and ankyrin repeat containing E3 ubiquitin protein ligase 1
hMLH1	mutL homolog 1
HOXA11	homeobox A11
HOXA9	homeobox A9
IER3	immediate early response 3
KCNH8	potassium voltage-gated channel subfamily H member 8
KRAS	KRAS proto-oncogene
L1TD1	LINE1 type transposase domain containing 1
LGALS4	galectin 4
LOX	ysyl oxidase
MAPK13	mitogen-activated protein kinase 1
MDM2	MDM2 proto-oncogene
MGMT	O6-methylguanine-DNA methyltransferase
MT1	metallothionein 1A
MT2	metallothionein 2
MTHFD2	methylenetetrahydrofolate dehydrogenase (NADP+ dependent) 2
MYO1G	myosin IG
p14ARF	alternate reading frame protein product of the CDKN2A locus
p16INK4a	cyclin-dependent kinase inhibitor 2A
PAX6	paired box 6
PR	Progesterone receptor
PTEN	phosphatase and tensin homolog
RARß	retinoic acid receptor beta
RASSF1	Ras association domain family member 1
RNF	ring finger protein
SPAG6	sperm associated antigen 6
SYK	spleen associated tyrosine kinase
THBS1	thrombospondin 1
TIMP3	TIMP metallopeptidase inhibitor 3
TLL2	tolloid like 2
TNFSF10D	Tumor necrosis factor receptor superfamily, member 10d
TP53	tumor protein p53
TUBB3	tubulin beta 3 class III
TUBB6	tubulin, beta 6 class V
ZAR1	zygote arrest 1
ZNF385D	zinc finger protein 385D
ZNF677	zinc finger protein 677
